# Strength and Electrostatic Discharge Resistance Analysis of Additively Manufactured Polyethylene Terephthalate Glycol (PET-G) Parts for Potential Electronic Application

**DOI:** 10.3390/ma17164095

**Published:** 2024-08-18

**Authors:** Julia Talecka, Janusz Kluczyński, Katarzyna Jasik, Ireneusz Szachogłuchowicz, Janusz Torzewski

**Affiliations:** 1 Institute of Optoelectronics, Military University of Technology, Gen. S. Kaliskiego 2, 00-908 Warsaw, Poland; julia.talecka@student.wat.edu.pl; 2 Institute of Robots & Machine Design, Faculty of Mechanical Engineering, Military University of Technology, Gen. S. Kaliskiego 2, 00-908 Warsaw, Poland; katarzyna.jasik@wat.edu.pl (K.J.); ireneusz.szachogluchowicz@wat.edu.pl (I.S.); janusz.torzewski@wat.edu.pl (J.T.)

**Keywords:** additive manufacturing, material extrusion, PET-G, tensile test, digital image correlation, electrostatic discharge resistance

## Abstract

Optoelectronic components are crucial across various industries. They benefit greatly from advancements in 3D printing techniques that enable the fabrication of intricate parts. Among these techniques, Material Extrusion (MEX) stands out for its simplicity and cost-effectiveness. Integrating 3D printing into production processes offers the potential to create components with enhanced electrostatic discharge (ESD) resistance, a critical factor for ensuring the reliability and safety of optoelectronic devices. Polyethylene terephthalate glycol-modified (PET-G) is an amorphous copolymer renowned for its high transparency, excellent mechanical properties, and chemical resistance, which make it particularly suitable for 3D printing applications. This study focuses on analyzing the mechanical, structural, and electrostatic properties of pure PET-G as well as PET-G doped with additives to evaluate the effects of doping on its final properties. The findings highlight that pure PET-G exhibits superior mechanical strength compared to doped variants. Conversely, doped PET-G demonstrates enhanced resistance to electrostatic discharge, which is advantageous for applications requiring ESD mitigation. This research underscores the importance of material selection and optimization in 3D printing processes to achieve desired mechanical and electrical properties in optoelectronic components. By leveraging 3D printing technologies like MEX and exploring material modifications, industries can further innovate and enhance the production of optoelectronic devices, fostering their widespread adoption in specialized fields.

## 1. Introduction

Optoelectronic components play a crucial role in various industrial sectors, facilitating advancements in telecommunications, medical technology, lighting, photography, and many other fields [1,2]. Given the complex structure of these components, the application of 3D printing techniques may be beneficial. Three-dimensional printing revolutionizes the production of small, intricate elements [3,4]. One of the most commonly used 3D printing methods is Material Extrusion (MEX), also known as Fused Deposition Modeling (FDM) or Fused Filament Fabrication (FFF) [5,6]. This technique uses thermoplastic polymer extrusion to create three-dimensional parts. Since its inception, MEX technology has become one of the most popular and recognizable additive manufacturing methods due to its ease of use and cost-effectiveness [7]. The use of this technology allows for the combination of Electrostatic Discharge Resistance (ESD) in polymer materials with appropriate additives while meeting the geometric requirements of optoelectronic components. Filament materials are readily available, and the extrusion process is precise, relatively inexpensive, and straightforward.

The authors of [8] produced diffusion tubes with Ion Mobility Spectrometry (IMS) using the MEX method. They demonstrated the possibility of single-run 3D printing of an IMS drift tube with integrated gate and aperture grids using a three-fiber 3D printing system, which provided better quality and less waste than traditional methods. The importance of testing printing parameters such as temperature and extrusion speed was also emphasized, as these affect the ESD conductivity of the filament and can yield different results on various 3D printers. Hauck et al. [9] designed a one-piece drift tube for IMS using 3D printing, consisting of alternating conductive and insulating layers. The goal was to increase the accuracy and repeatability of the measurements compared to traditional drift tubes assembled manually, which can introduce errors in calculating mobility. The results showed that the 3D printed drift tubes had uniform lengths and minimal weight differences, leading to accurate and repeatable calculations. It was confirmed that 3D printing could be an effective method for producing drift tubes of consistent length, thus improving measurement precision in IMS. Su et al. [10] developed a method for fully 3D printing flexible Organic Light-emitting Diode (OLED) displays. Through a multimodal printing approach that combines extrusion and spraying methods, they constructed devices with significantly improved uniformity of active layers and more stable polymer–metal connections. Spray printing, which was used to deposit active layers, improved their uniformity by reducing the directional mass transport. Additionally, mechanical reconfiguration of the liquid metal surface increased the contact area of the polymer–metal connections. The patent [11] describes an advanced LED lighting device that provides improved convergence of light beams and increased radiation range compared to traditional reflectors. The design includes a high thermal conductivity base with a curved upper portion, an internal container with a flat mounting surface, and a curved shield with an internally reflective surface. These components enable the focusing of LED light beams and minimize dispersion, significantly enhancing the device’s lighting efficiency.

In the context of optoelectronic devices, there is a risk of ESD, which poses a threat to both operators and the devices themselves [12,13]. Phenomena such as separation and induction can generate excess electrostatic charges, which can lead to damage, especially in devices sensitive to ESD [14,15]. The electronics industry has numerous standards that define methods for controlling, testing, and taking preventive actions to ensure the safe handling of electronic components, including during production. Using materials with adequate surface resistance and good mechanical properties to manufacture structural components and enclosures of optoelectronic devices is crucial to ensuring the proper functioning and safety of these devices and their users [9]. The patent [16] presents innovative applications of electrostatic discharge-resistant enclosures, particularly for optoelectronic devices. One example of a patented enclosure includes a reflecting element designed to both reflect electromagnetic radiation emitted by the semiconductor device and absorb electromagnetic radiation directed toward the device. The reflecting part is covered with the same material as the rest of the enclosure, but the internal reflective part remains partially uncovered. The reflecting element is made of a different type of synthetic material than the rest of the enclosure, differing in at least one significant material property, such as thermal stability or resistance to electromagnetic radiation. A key aspect of the design is that the reflecting part is not connected to the rest of the enclosure using adhesives or mechanical macroscopic connections but, rather, joins directly through contact. The patent also presents a series of other solutions related to various materials and structural elements of enclosures used in different applications.

The polymers most commonly used in electronic applications are Acrylonitrile Butadiene Styrene (ABS), Polylactic Acid (PLA), Polyetheretherketone (PEEK), Polybutylene Terephthalate (PBT), and polyethylene terephthalate glycol-modified (PET-G) [17,18,19,20,21,22,23,24].

Polyethylene terephthalate glycol-modified (PET-G) is an amorphous copolymer with good transparency, mechanical properties, and chemical resistance, which is used in 3D printing. It is characterized by high mechanical strength and thermal stability. PET-G shows better thermal degradation and higher thermal stability compared to other materials used in 3D printing, making it suitable for producing components that require high strength and flexibility [25,26]. The patent [11] proposes an advanced optoelectronic device utilizing a housing made from two different types of plastics with specific properties. The first material, such as polybutylene terephthalate (PBT), PET-G, or polyetheretherketone (PEEK), is chosen for its high resistance to electromagnetic radiation and thermal stability. The second material, such as polyamides or polyphenylene sulfone, is selected for other structural aspects. The reflecting part of the housing is made from the first type of material, to which a white pigment like titanium dioxide is added to enhance its ability to reflect radiation. The enclosure provides a suitable environment for optoelectronic components such as light-emitting diodes (LEDs) or lasers that emit electromagnetic radiation. These elements are mounted in the housing in a manner that allows controlled reflection or scattering of radiation. The use of materials with increased resistance to electrostatic discharge, such as PET-G, enables the implementation of this solution using additive manufacturing methods, potentially increasing production flexibility and efficiency.

Given the benefits of PET-G compared to other widely used 3D printing materials, this study aims to investigate selected strength properties of PET-G and evaluate whether doping the material positively affects its final properties. There is a lack of available research results of this type; therefore, the novelty lies in compiling strength and structural analysis results with surface resistance data. This allows us to address whether the additively manufactured component will provide adequate strength while maintaining ESD resistance.

## 2. Materials and Methods

### 2.1. Materials for the Research

The first material selected for research was the ESD-resistant PET-G filament. This material is relatively easy to print with and does not require a heated chamber. The manufacturer claims that the filament is resistant to electrostatic discharge. The next material chosen was pure PET-G, which was selected to investigate whether the addition of compounds improves the properties of the final filament. The last material selected was PET-G doped with carbon fiber (up to 10% carbon fiber content). This material has high mechanical strength and is designed to operate in extreme temperatures. It is an industrial-grade filament that can be processed and used on 3D printers. It is safe for both humans and the environment, as it does not emit toxic fumes and exhibits minimal shrinkage. This selection of materials allowed us to compare and assess how doping affects the final mechanical and electrostatic properties of PET-G. All materials were supplied by Spectrum Filaments (Spectrum Filaments, Pęcice, Poland).

### 2.2. Manufacturing Process

**Figure 1:**
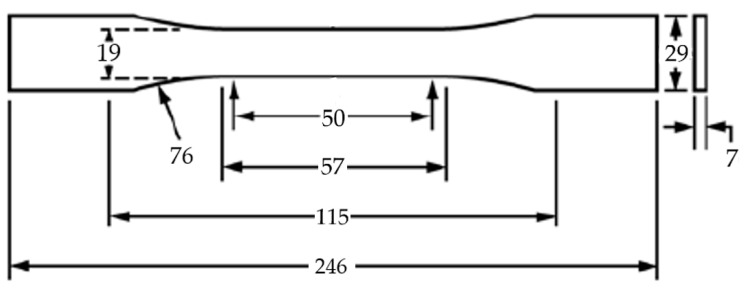
Dimensions of the tensile test specimen [27].

The 3D models of the test samples were designed using SolidWorks CAD software (Dassault Systems; Waltham, QC, Canada) (version 2023). Dogbone-shaped samples were designed for static tensile strength according to the relevant standard ASTM D638-14:2022—Standard Test Method for Tensile Properties of Plastics [27] (Figure 1).

Rectangular samples measuring 200 mm × 200 mm × 3 mm for surface resistance testing were designed according to the appropriate standards [28].

**Figure 2:**
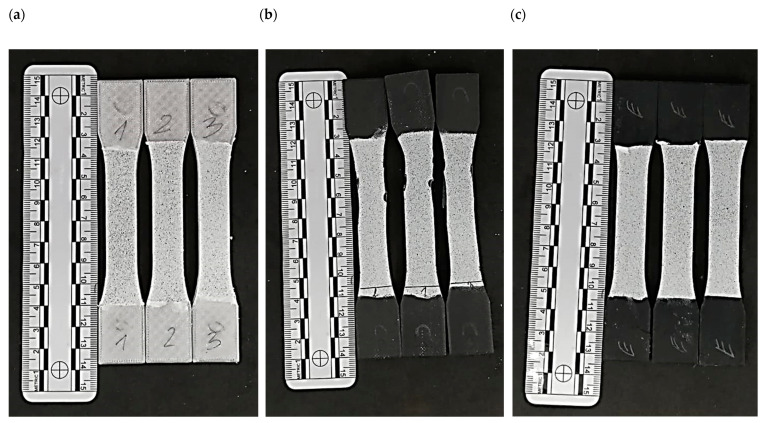
Printed models of samples, prepared for testing: (**a**). PET-G; (**b**). Carbon fiber PET-G; (**c**). ESD PET-G.

The test samples were fabricated using the Material Extrusion (MEX) technique. The manufacturing process was carried out using a Prusa i3 MK3s 3D printer (Prusa Research, Prague, Czech Republic). The print codes were prepared in advance using the dedicated 3D printing software Prusa Slicer v2.5.2. The process parameters for all types of samples were kept consistent and are detailed in Table 1. Figure 2 shows printed samples. 

### 2.3. Testing Methods

The static tensile test of the fabricated samples was performed according to the standard ASTM D638-14:2022—Standard Test Method for Tensile Properties of Plastics [27]. Experiments were performed using a classic INSTRON 8802MTL tensile testing machine (Norwood, MA, USA) equipped with WaveMatrix software (version 2.0) (Instron, Norwood, MA, USA). Strain measurements were obtained using an extensometer (2620-604, INSTRON, Norwood, MA, USA) with a gauge length of 50 mm. Furthermore, the properties were examined by surface analysis using Digital Image Correlation (DIC).

Subsequently, the surface structure of the samples was analyzed after tensile testing using a Keyence VHX-7000 digital optical microscope (Keyence, Osaka, Japan). Representative samples of each material were selected for this analysis.

Following this, the resistance to electrostatic discharge was evaluated using the relevant standard. Resistance measurements were carried out using an Aijgo 61 resistance meter (Aijgo, Vác, Budapest). The tests were carried out in a laboratory setting after conditioning the samples for 48 h at a temperature of 23 ± 2 °C and a relative humidity of 12 ± 3% RH. Surface resistances were measured using various Concentric Ring Electrodes (CRE) following the standard guidelines. To reduce contact resistance, measurements were repeated using a Surface Resistivity Bar (SRB) electrode. The SRB electrode had a mass of 2900 g, a bar width of 3 mm, and a length of 50.8 mm, with a spacing of 25.4 mm between the bars. The contact surface was made of conductive rubber (3 mm thick, hardness A 60, ρV < 100 Ωm) or copper. Dimensions were considered, and the results were expressed as resistance.

Material resistance was measured using an electrode with a mass of 2.3 kg and a diameter of 63 mm. Various types of conductive rubber were compared to determine the lowest possible contact resistance between the upper and lower electrodes and the sample. As a result of the unstable contact between the electrode and the samples, concentric ring electrodes (CREs) could not be used. Furthermore, DC resistance through the material was measured using an ESD contact probe with a rounded tip. Results were recorded after 15 s of electrification. The test voltage was 10 V for resistances below 1 MΩ or 100 V for resistances greater than 1 MΩ. All measurements were repeated ten times, with the results presented as minimum and maximum readings along with the geometric mean.

## 3. Results

All tests were performed on samples printed on three different materials. The descriptions of the results of all tests correspond to the classifications described in Table 2. 

### 3.1. Static Tensile Test 

**Figure 3:**
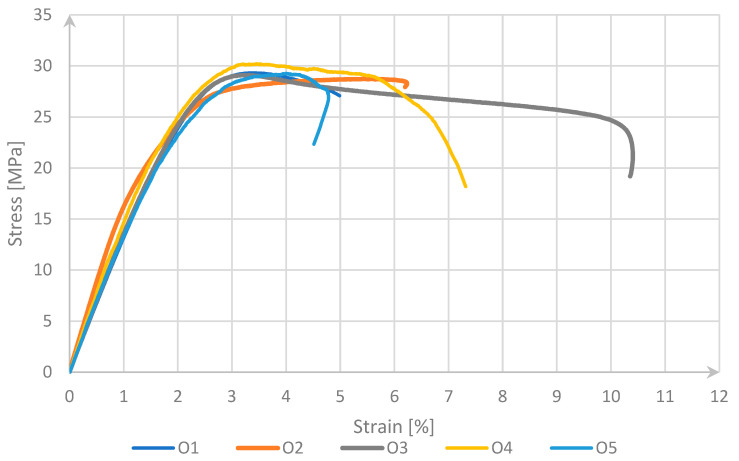
Stress–strain curve for PET-G.

**Figure 4:**
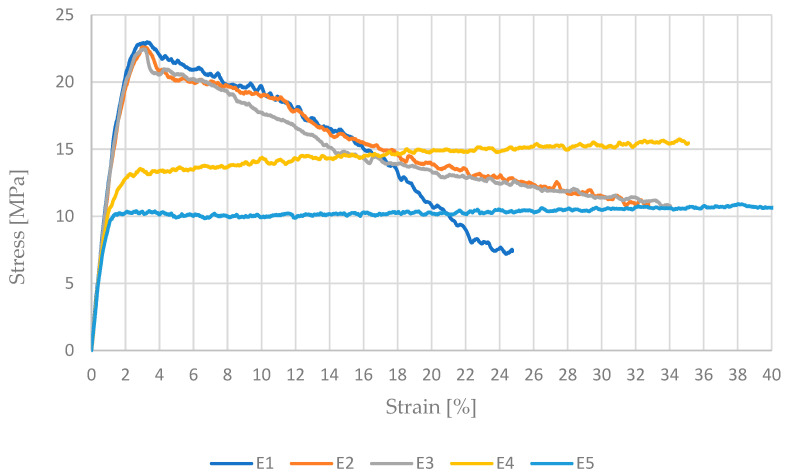
Stress–strain curve for ESD PET-G.

**Figure 5:**
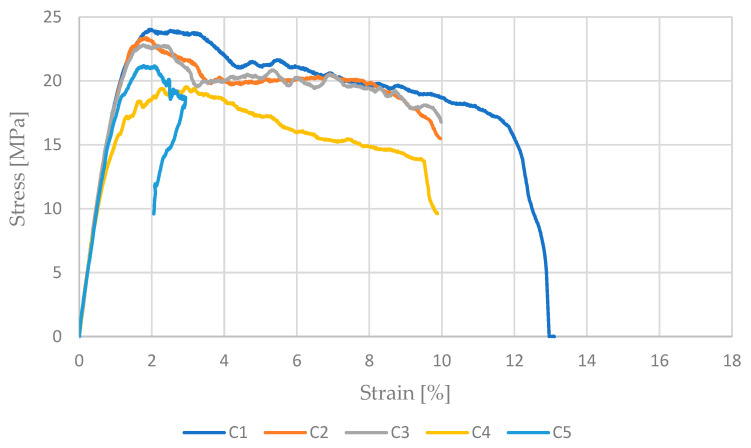
Stress–strain curve for PET-G carbon fiber.

To determine the tensile strength, five measurements were made for each type of sample. The results obtained are presented in Figure 3, Figure 4 and Figure 5. While analyzing the graph for undoped PET-G presented in Figure 3, it was observed that the obtained samples are stable but lower in comparison to the results presented in articles such as [29]. Such a phenomenon could be related to the different suppliers of the material that could use different base materials for filament production. All the specimens also exhibited stress results below the standard, approximately 30 MPa. The likely cause of these differences is the use of the same manufacturing parameters for all material configurations. The curve selected for comparison was identified from all the tests in which the stress values were around 30 MPa, aligning with the results presented in other scientific studies on this material, such as [29]. Subsequently, tests were conducted on materials with additives enhancing electrostatic discharge (ESD) resistance. In the case of PET-G samples marked with the symbol O, one type of fracture mechanism occurred. All samples carried the highest load with a deformation in the range of 4–7%. This is due to the good cohesion of the external layer with the internal structure of the sample.

The plotted graph (Figure 4) for the ESD material showed a significantly reduced maximum stress value compared to the reference material. The doping resulted in a decrease in strength to approximately 23 MPa. The graph showed two curves that significantly deviated from the rest. Hence, the first three samples exhibiting the highest tensile strength were taken as the standard. Considering the results presented in the article [30], it can be noted that they are significantly better, resembling more closely the values for undoped PET-G. The inferior properties of PETG ESD compared to the results of the study may be due to the use of two different manufacturers for this type of material. The material datasheet tested during the static tensile test does not provide information on the elements used during doping. Another reason for the reduced stress value could be the printing parameters. Additionally, an important factor is the fill pattern. The researchers in the article [30] applied values for several fill patterns, yielding different strength results ranging from approximately 34 to 51 MPa. The lowest result corresponded to the circular fill, while the highest was achieved with the linear fill pattern. In the case of samples marked “E”, samples E1, E2, and E2 were considered representative and correctly made. The compact structure of the outer layer and the core ensured high strength and a gentle course of strength loss. In the case of samples E4 and E5, the internal structure mainly transferred elastic loads. After exceeding the yield point, the load was taken over by the outer layer. It gradually narrowed locally. This narrowing gradually propagated along the entire length of the sample.

Subsequently, samples made from carbon fiber-reinforced filament were tested. The graph in Figure 5 shows the stress–strain curves for PET-G reinforced with carbon fiber, which were the most consistent among all the tested samples in this series. The maximum values ranged from 21 MPa to 24 MPa. In studies published in the article [31], results around 30 MPa were reported, suggesting that the tests were conducted properly and the obtained results are comparable. For PET-G CF, the first three samples, whose curves almost overlapped, were considered representative. Given that all specimens, regardless of material type, were printed using the same parameters, it can be concluded that the applied additives significantly influence the strength properties of the printed elements. PET-G carbon fibre samples. Samples C1–C4 were characterized by good repeatability. Carbon fibers ensured a good connection of the elastic material and its cohesion in the range of plastic deformations. Sample C5 transferred the maximum load at an acceptable level, but the level of deformations was low. This resulted from the increased concentration of carbon fibers in a small area, which caused a dynamic fracture of the sample.

**Figure 6:**
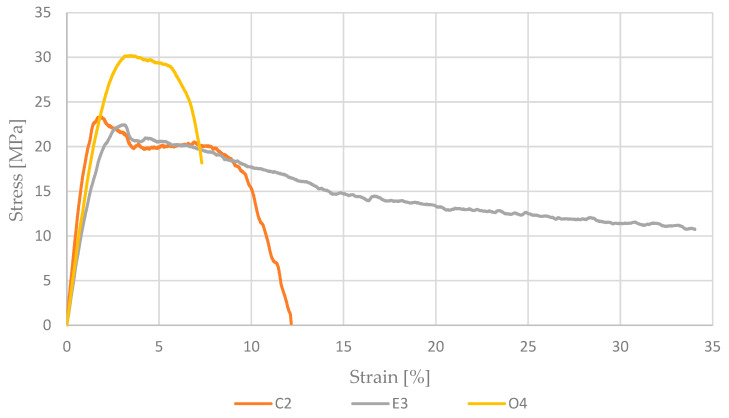
Representative stress–strain curves for all sample series.

Figure 6 illustrates representative curves for all materials used in the strength tests. Due to the large scatter of results in mechanical tests, the authors selected curves for samples with the highest tensile strength. This criterion allows for the presentation of fracture mechanisms for samples carrying the highest load, during which the weakest links in the manufacturing process are clearly revealed.

The C2 sample achieves a maximum stress of about 23 MPa at a strain of around 6%, followed by a rapid decline in stress to near zero at a strain of about 10%. The E3 sample reaches a maximum stress of approximately 19 MPa at a strain of around 3%, and then the curve declines and remains relatively stable at around 15 MPa until a strain of about 35%. The O4 sample reaches a maximum stress of about 25 MPa at a strain of around 5%, followed by a gradual decline in stress to near zero at a strain of about 12%. Each of the tested materials exhibits different behavior and achieves different mechanical properties. The E3 sample shows the greatest ability to deform at relatively constant stress after reaching the maximum value, while the C2 and O4 samples exhibit distinct drops in stress after reaching their maximum values, with the C2 sample showing the fastest decline. The parameters used for printing in MEX technology significantly influenced the obtained results. Additionally, the storage conditions of the filaments may also be a factor contributing to lower strength results, with the addition of carbon fibers potentially increasing the material’s water absorption.

### 3.2. Digital Image Correlation 

**Figure 7:**
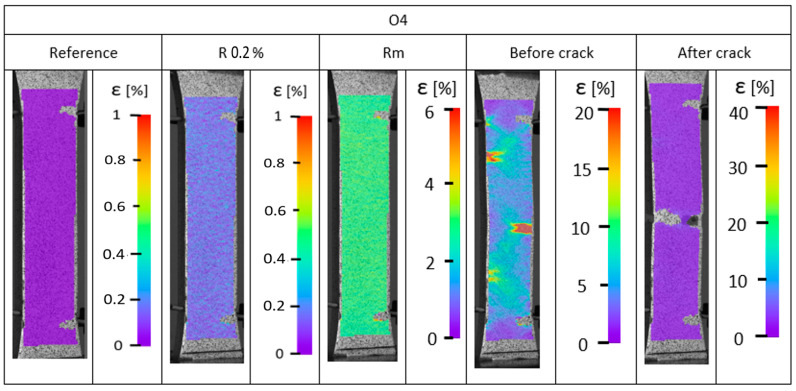
Strain distribution recorded using digital image correlation for PET-G.

**Figure 8:**
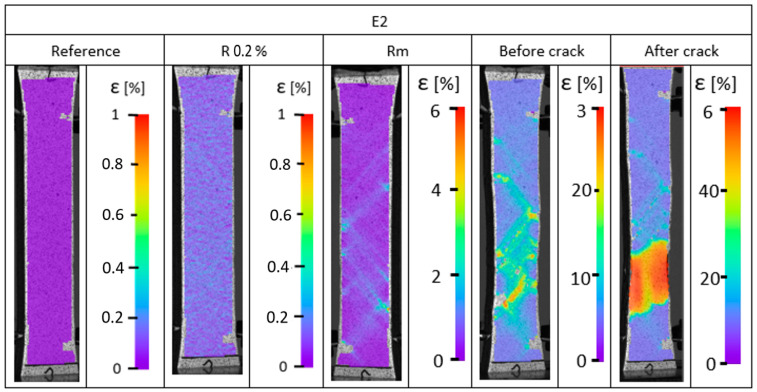
Strain distribution recorded using digital image correlation for EDS PET-G.

**Figure 9:**
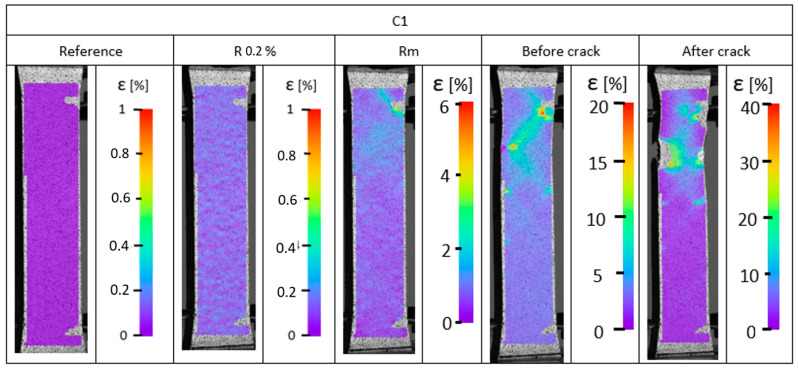
Strain distribution recorded using digital image correlation for carbon fiber PET-G.

During the static tensile test, strain analysis was conducted. The strain distributions are presented in Figure 7, Figure 8 and Figure 9. The results obtained indicate a heterogeneous strain distribution on the surface of all the samples analyzed. This is particularly evident for pure PET-G (Figure 7), where two distinct areas of higher strain were observed after exceeding the yield point. This phenomenon may be due to inaccuracies in the print structure, where crack initiation likely occurred at these locations because of weak connections between the material’s outline and the infill. Digital image correlation confirmed the tensile test results.

The doped materials (Figure 8 and Figure 9) exhibited much greater ductility, as measurements were taken only up to the end of the extensometer range rather than until the sample fracture. Consistently, for samples made of PC/PET-G (Figure 9) and PET-G ESD (Figure 8), areas of reduced tensile resistance were observed and identified as crack initiation points. For all samples, these regions are located near the boundary of the sample and propagate along the infill lines of the model. The propagation is attributed to the anisotropy of the mechanical properties, which indicates the variation of resistance in different directions, which is directly related to 3D printing technology.

For all samples tested, particularly just before fracture, linearly arranged areas (at an angle of approximately 45°) are observed with increased strain. This phenomenon is associated with the change in the orientation of layer deposition during the 3D printing process, which is another factor influencing the degradation of elements manufactured using PET-G-based materials.

### 3.3. Analysis of Fracture Surfaces after Static Tensile Testing

**Figure 10:**
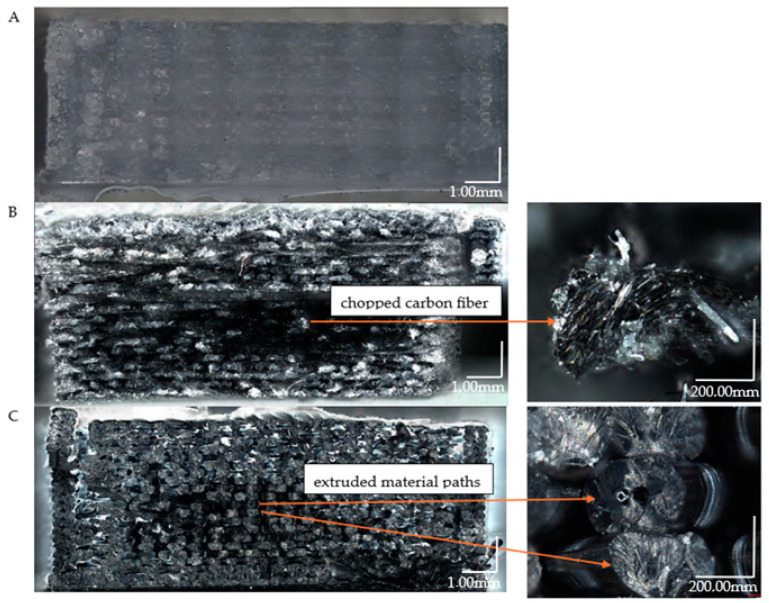
Sample fracture structures after static tensile testing for the following: (**A**) PET-G; (**B**) Carbon fiber PET-G; (**C**) EDS PET-G.

We show the results of the surface fracture analysis following the static tensile test, which was conducted for the cross-sections of the three types of samples examined (Figure 10A–C). The structure of pure PET-G (Figure 10A) is the most solid, with individual paths of deposited material fused together without distinct gaps. In the case of doped samples (Figure 10B,C), the individual paths of the printed material are visible, and single fibers used to dope the material can also be observed.

**Figure 11:**
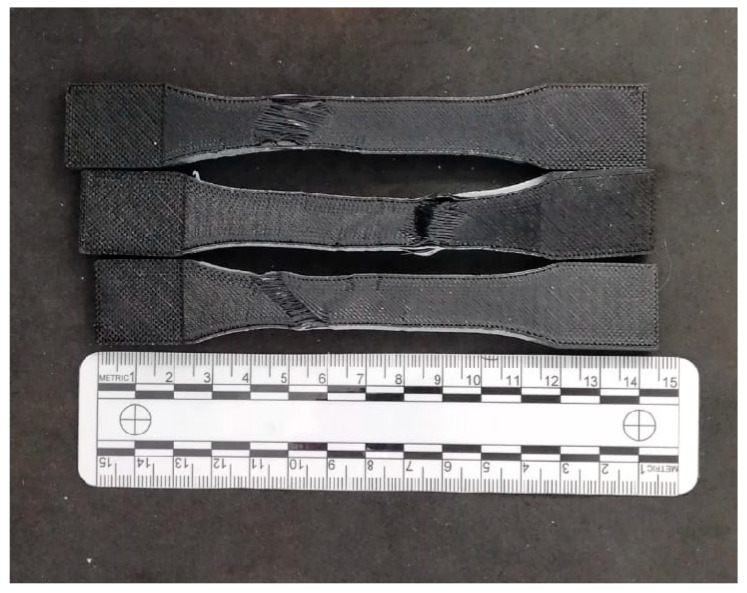
Samples after static tensile testing made of carbon fiber PET-G.

In the structures examined by means of a microscope for all samples, fractures predominantly exhibit brittle cracking. In some areas, plastic cracks are observed. The initiation points of material failure are also clearly visible. The first sample examined is pure PET-G (Figure 10A). The crack initiation point is marked in the close-up. The microscopic analysis also revealed that the surface in direct contact with the bed during printing has fibers much more tightly bonded than those of the upper layers. This phenomenon occurred in both pure PET-G and doped variants (Figure 10B,C). A difference in structure is visible between the wall and the filling. The second sample (Figure 10B) contained carbon fibers. It is noticeable that during the tensile test, the fibers were separated from the PET-G matrix, which has been confirmed in other studies [32]. Individual layers are not well bonded, and the broken carbon fibers create voids in the material structure. The strength of individual fibers affects the overall sample strength. In specimens with this additive, the direction in which the 3D printer places the infill negatively impacted the results. The best effects could be achieved with lines parallel to the tensile force. The destruction of material occurred over a large area of the sample. The course of the fracture process of the internal structure is unchangeable. Only the external structure has a random fracture course, and additionally, the authors wanted to present this in Figure 11. 

The last material tested was PET-G ESD (Figure 11), which exhibited the least cohesive fracture. In the microscopic image of the sample, large gaps in the central infill are noticeable. As with the other materials, the best bonding was observed between the layers printed on the bedside and those forming the walls. These structures influenced the final strength properties. The bottom surface of the sample also contacted the adhesive applied to the bed during printing. Due to the elevated temperature (approximately 60 °C), the adhesive likely bonded with the filament material. Close-ups reveal areas characteristic of plastic cracking. Unlike the other materials, the fibers in PET-G ESD mainly carried the load instead of breaking brittlely. Additionally, fibers with internal voids were observed, which could have significantly impacted the results obtained during the static tensile test.

### 3.4. Electrostatic Resistance

The results of the tests conducted on electrostatic discharge resistance for the printed samples are presented in Table 3. 

The surface resistivity values for samples made of PET-G ESD confirm their resistance to electrostatic discharge. The resistance test results align with observations made under the microscope. A sample labeled E did not meet the ESD resistance requirements from the workbench side. The result within the limit is over 1000 times smaller. One possible reason for the difference between the bottom and top layers of the PETG ESD material sample could be the use of an additional adhesive substance. Due to the nature of 3D printing, the temperature on the bed during the process is 60 °C. The adhesive substance in direct contact with the first layer of the model may penetrate the material structure and alter its properties. For applications requiring ESD resistance, it would be advisable not to use such substances or to pre-treat the prototype surface to remove adhesive residues. In addition, parameters such as sample thickness and infill type can be significant. Depending on the application, it may be necessary to perform additional measurements to determine the ESD resistance values for the final models. 

## 4. Conclusions

In this study, selected mechanical strength, structural, and electrostatic tests were performed on selected materials for optoelectronic applications. The aim was to understand the behavior of additively manufactured materials under mechanical load, including static tensile testing using DIC, fracture microstructure analysis, and their ESD. The results of these tests led to the following conclusions:

- PET-G without additives achieved the highest stress values (30 MPa), while ESD additives and carbon fibers reduced the strength to 23 MPa.
- PET-G ESD exhibited significant plasticity, reaching an elongation at a break of 32%, which is four times the breaking strain of pure PET-G.
- DIC analysis allowed for a detailed examination of surface deformations, showing greater plasticity in materials with additives and increased tensile resistance.
- Fracture microstructure analysis identified crack initiation sites and specific fracture features in doped materials.
- Surface resistance tests confirmed that PET-G ESD effectively disperses electrostatic discharges.

These findings underscore the dual benefits of PET-G in additive manufacturing: pure PET-G excels in mechanical strength, while doped PET-G formulations exhibit enhanced plasticity and superior ESD dissipative properties. This dual capability positions PET-G as a versatile material for a wide range of optoelectronic applications, from structural components requiring high mechanical integrity to devices demanding stringent ESD protection.

## Figures and Tables

**Table 1:** Printing parameters of the MEX.

Filament Diameter [mm]	Nozzle Diameter [mm]	Printing Temperature [°C]	Bed Temperature [°C]	Print Speed [mm/s]	Infill Pattern	Infill [%]	Number of Contours
1.75	0.4	230	80	70	linear	100	3

**Table 2:** Descriptions of the series of samples used during the research.

Material Condition	Specimen Description
PET-G	O
Carbon fiber PET-G	C
ESD PET-G	E

**Table 3:** Surface resistivity measurement results for the samples.

Tested Samples	Results on the Upper Surface		Results on the Lower Surface (Build Plate Side)	
	Voltage Load 100	Conclusion	Voltage Load 100	Conclusion
Samples O	over limit	material is an isolator	over limit	material is an isolator
Samples E	8.7 × 10^6^ Ω	material exhibits dissipative properties	17 × 10^9^ Ω	material does not exhibit dissipative properties— beyond ESD limit
Samples C	over limit	material is an isolator	over limit	material is an isolator

## Data Availability

The raw data supporting the conclusions of this article will be made available by the authors on request.
